# Cell Expansion-Mediated Organ Growth Is Affected by Mutations in Three *EXIGUA* Genes

**DOI:** 10.1371/journal.pone.0036500

**Published:** 2012-05-04

**Authors:** Silvia Rubio-Díaz, José Manuel Pérez-Pérez, Rebeca González-Bayón, Rafael Muñoz-Viana, Nero Borrega, Gregory Mouille, Diana Hernández-Romero, Pedro Robles, Herman Höfte, María Rosa Ponce, José Luis Micol

**Affiliations:** 1 Instituto de Bioingeniería, Universidad Miguel Hernández, Campus de Elche, Elche, Alicante, Spain; 2 Institut Jean-Pierre Bourgin, UMR1318 Institut National de Recherche Agronomique/AgroParisTech, INRA Centre de Versailles-Grignon, Versailles, France; Instituto de Biología Molecular y Celular de Plantas, Spain

## Abstract

Organ growth depends on two distinct, yet integrated, processes: cell proliferation and post-mitotic cell expansion. Although the regulatory networks of plant cell proliferation during organ growth have begun to be unveiled, the mechanisms regulating post-mitotic cell growth remain mostly unknown. Here, we report the characterization of three *EXIGUA* (*EXI*) genes that encode different subunits of the cellulose synthase complex specifically required for secondary cell wall formation. Despite this highly specific role of *EXI* genes, all the cells within the leaf, even those that do not have secondary walls, display small sizes in the *exi* mutants. In addition, we found a positive correlation between cell size and the DNA ploidy levels in *exi* mutant leaves, suggesting that both processes share some regulatory components. Our results are consistent with the hypothesis that the collapsed xylem vessels of the *exi* mutants hamper water transport throughout the plant, which, in turn, limits the turgor pressure levels required for normal post-mitotic cell expansion during leaf growth.

## Introduction

The final size of plant organs is achieved by the rate of two distinct but integrated processes, cell proliferation and post-mitotic cell expansion, which increase the cell number and cell size, respectively [1]. However, the shape of the plant cell is an important determinant of plant morphogenesis, which is firmly controlled by the organization of the cell wall [2]. Leaves are determinate lateral organs that arise from the flanks of the shoot apical meristem (SAM) as dome-shaped structures, which grow by laminar expansion up to a certain size [3], [4]. To identify the genetic components involved in leaf morphogenesis, several genetic screens for non-lethal and visible mutations that disrupt the shape and size of Arabidopsis leaves have been performed [5]–[7]. To date, dozens of these leaf mutants have been characterized at the phenotypic and molecular levels, and these studies have revealed that the wild-type products of the genes involved participate in such diverse processes as polar cell expansion, the transduction of hormonal signals, gene regulation, plastid biogenesis, and chromatin remodeling [8].

Recent work has provided evidence for the organ-wide coordination of cell proliferation and expansion. In Arabidopsis leaves, a decrease in cell number below a certain threshold triggers an increase in mature cell size, a phenomenon that has been termed “compensation” [9], [10]. In *angustifolia3* (*an3*) mutants, which are impaired in cell proliferation, the palisade mesophyll cells are larger than normal [11], and *AN3* is thought to act as a positive regulator of cell proliferation in leaves [11]. Additional loss-of-function mutants exhibiting a compensation phenotype in their palisade mesophyll cells have been identified and named *fugu1* to *fugu5* [12]. Examination of the *fugu* mutants showed that compensated cell enlargement does not occur via the uncoupling of cell division and cell growth, rather, it is sustained by the specific upregulation of cell expansion [12]. Using a Cre/lox recombination system designed for the clonal activation and deletion of *AN3*, *an3*-dependent compensation was shown to be a non-cell-autonomous process, and *an3* cells seemed to generate and transmit an intercellular signal that enhances post-mitotic cell expansion in neighboring cells [13]. On the other hand, the *extra-small sisters* (*xs*) mutants are specifically impaired in cell expansion and are likely to be affected in the cell enlargement response during compensation [14]. Some of these *xs* mutations strongly suppress the compensated cell enlargement phenotype that is triggered by *an3* [14]. This finding led to the conclusion that compensated cell enlargement is mediated by the hyperactivation of cell expansion pathways that require the *XS* genes.

To identify additional factors involved in the coordination of leaf growth, we selected four *exigua* (*exi*) mutants from our EMS-induced leaf mutant collection that display small leaves with relatively normal shapes [5]. We found that these *exi* mutants exhibited specific defects in cell expansion but normal palisade mesophyll cell numbers. The *exi* mutations were found to affect three genes encoding three subunits of the cellulose synthase complex that is required for secondary cell wall biosynthesis: CESA4, CESA7, and CESA8 [15]. We gathered empirical data to provide a causal explanation that accounts for the small leaf phenotype of the *exi* mutants. Our results are consistent with the hypothesis that internal turgor pressure drives cell expansion during leaf growth. Based on our own study and those of others, we hypothesize that a turgor-mediated cell expansion mechanism might account, at least in part, for leaf growth coordination in Arabidopsis.

## Results

### The *Exigua* Mutants Display Small Leaves Due to Defective Cell Expansion

**Figure 1:**
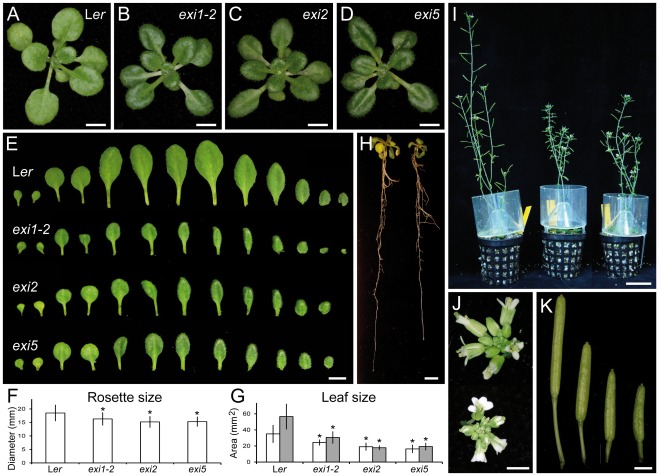
Morphology of the *exi* mutants. (A–D) Rosettes of the (A) Landsberg *erecta* (L*er*) wild type and the (B) *exi1-2*, (C) *exi2* and (D) *exi5* mutants. (E) Series of L*er*, *exi1-2*, *exi2* and *exi5* leaves. From left to right: two cotyledons and the first to tenth or eleventh leaves. Mutants display small, dark-green leaves of relatively normal shape. (F, G) (F) Rosette mean diameter and (G) lamina area from first-node (white bars) or third-node (grey bars) leaves. Asterisks indicate significant differences from the L*er* control (*P* value<0.05; n = 11–20 individuals). (H) Root morphology in L*er* (left) and *exi5* (right) seedlings grown in near-vertically oriented plates. (I) From left to right, the reproductive morphology of L*er*, *exi1-2* and *exi2* plants grown in pots. (J) Detail of L*er* (up) and *exi1-2* (down) inflorescences. (K) From left to right, siliques from L*er*, *exi1-2*, *exi2* and *exi5* plants. Samples were taken (A–H) 21 and (I–K) 38 DAS. All the plants shown were homozygous for the indicated mutations (in italics). Scale bars indicate (A–E, H, J and K) 5 mm and (I) 3 cm.

In a forward genetic screen, we previously identified hundreds of mutations affecting the size and shape of the vegetative leaves of *Arabidopsis thaliana* [5]. Four of the recessive mutations from this collection, *exigua1-1* (*exi1-1*), *exi1-2*, *exi2* and *exi5*, caused a phenotype that is characterized by small, dark-green vegetative leaves of a relatively normal shape (Figure 1A-E). First- and third-node leaf area was significantly reduced in the *exi* mutants in comparison with their wild-type, Landsberg *erecta* (L*er*) (Figure 1E and 1G). Other organs in the *exi* mutants, such as the primary root (Figure 1H) and the main inflorescence stem (Figure 1I), displayed smaller sizes than those in L*er*. The flowers (Figure 1J) and mature siliques (Figure 1K) of the *exi* mutants were also reduced in length compared with L*er*.

**Figure 2:**
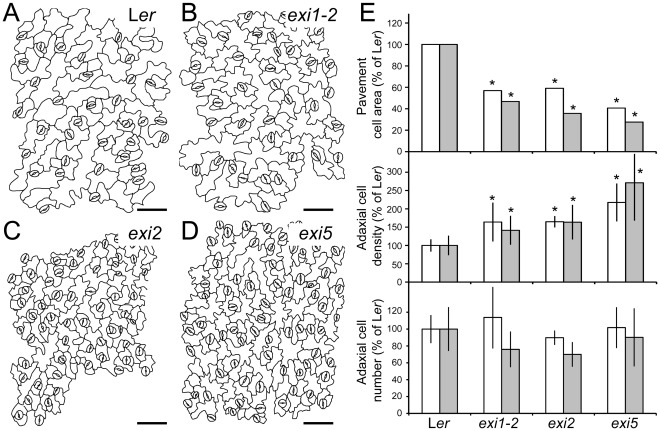
Leaf adaxial epidermis in the *exi* mutants. (A–D) Representative diagrams from the leaf adaxial epidermis of *exi* mutants. Diagrams were drawn from the differential interference contrast images of cleared third-node leaves collected from (A) L*er*, (B) *exi1-2*, (C) *exi2* and (D) *exi5* plants. (E) Adaxial pavement cell area, cell density and cell number in first-node (white bars) or third-node (grey bars) leaves of the *exi* mutants. Data were normalized with respect to the L*er* value and are expressed as percentages. Represented values are the mean and the standard deviation, except for the pavement cell area, which did not exhibit a normal distribution and for which the median was used. Asterisks indicate significant differences from the L*er* control (*P* value<0.05; n = 11–15). All the data were obtained at 21 DAS from plants grown on half-strength MS agar medium. Scale bars indicate 50 µm.

**Figure 3:**
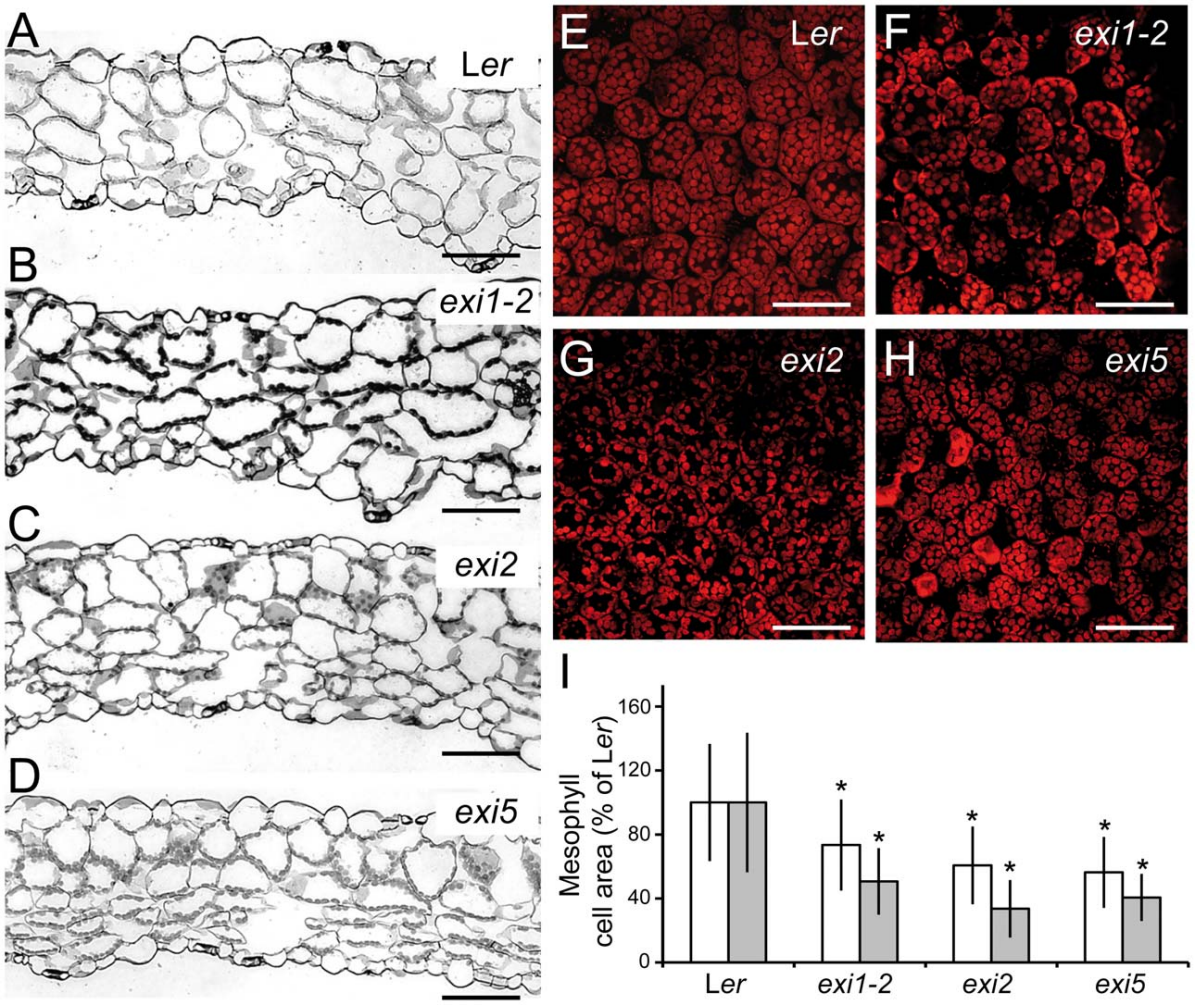
Internal leaf structure in the *exi* mutants. (A–D) Transverse sections of a region midway between the midvein and the leaf margin from (A) L*er*, (B) *exi1-2*, (C) *exi2* and (D) *exi5* third-node vegetative leaves. (E–H) Confocal micrographs of palisade mesophyll sections of first-node leaves from (E) L*er*, (F) *exi1-2*, (G) *exi2* and (H) *exi5* mutants. (I) Palisade mesophyll cell size in the subepidermal layer of first-node (white bars) or third-node (grey bars) leaves of L*er* and *exi* mutants. Data were normalized with respect to the L*er* value and expressed as percentages (mean and standard deviation). Asterisks indicate significant differences from the L*er* control (*P* value<0.05; n = 13–16 individuals). All the data were obtained at 21 DAS from plants grown on half-strength MS agar medium. Scale bars indicate (A–D) 100 µm and (E–H) 50 µm.

To describe the leaf phenotype of the *exi* mutants at the cellular level, we drew the cell borders from differential interference contrast (DIC) micrographs of the adaxial epidermis and the palisade mesophyll cells (see Materials and Methods). In the leaf epidermis of first- and third-node leaves, the sizes of the pavement cells and guard cells are significantly reduced in the *exi1-2*, *exi2* and *exi5* mutants compared with L*er* (Figure 2). The number of epidermal cells per leaf lamina, as estimated from the average values for adaxial epidermal cell densities and lamina areas (see Materials and Methods), do not significantly differ between the *exi* mutants and L*er* (Figure 2E), indicating that the *exi* mutations do not alter leaf epidermal cell divisions. We also studied internal leaf structure in the *exi* mutants (Figure 3). Transverse sections of the third-node vegetative leaves of *exi1-2*, *exi2* and *exi5* homozygotes did not reveal significant differences in the interveinal regions (Figure 3A–D). However, using confocal laser-scanning microscopy of native chlorophyll fluorescence and assuming that the number of chloroplasts per palisade mesophyll cell is similar in all the genotypes studied (Figure 3E–H), we found that the cell size is significantly reduced in the *exi1-2*, *exi2* and *exi5* mutant leaves in comparison with those in L*er*. In addition, the sizes of the mesophyll cells, measured using differential intereference contrast micrographs, were significantly reduced in the first- and third-node leaves of the *exi* mutants compared with L*er* (*P* value<0.05) (Figure 3I). Taken together, our results indicate that the recessive *exi* mutations specifically reduce the size of every cell within the leaf, which in turn generates small leaves with a dark-green color due to an increased chloroplast density in their mesophyll cells. The morphological phenotype of different organs in the *exi* mutants suggests a positive role for the *EXI1*, *EXI2* and *EXI5* genes in the regulation of cell expansion.

To investigate the functional relationship between the *EXI* genes, we obtained the *exi2 exi5* double mutant. Double mutant plants were dwarf and had small, dark-green leaves and short inflorescences (Figure S1), a phenotype that was stronger than those of their parentals. Based on the severity of this phenotype [16], we considered the *exi2 exi5* double mutant phenotype as additive.

### The *Exi* Mutations Affect Leaf Ploidy Levels

A positive correlation between cell size and nuclear ploidy levels has been found in wild-type Arabidopsis leaves [17], and we questioned to what extent the cell expansion defects observed in the *exi* mutant leaves might be caused by altered ploidy levels in their cells. Cells with higher ploidy levels (>4C) arise by endoreduplication (i.e., successive DNA replication cycles without intervening mitoses) [18]. We found that the 8C and 16C nuclei populations are significantly reduced in the first pair of leaves of the *exi* mutant rosettes compared to L*er* (Table 1), which is consistent with a global reduction of the endoreduplication levels in *exi* leaves. In addition, a mild but significant increase in the diploid nuclei population (2C) of the *exi* leaves compared with that of L*er* was found (Table 1). The 4C population was also more represented in the *exi* mutants, compared to L*er*, and includes both mitotic diploid cells in the S/G_2_ phase of the cell cycle and tetraploid endoreduplicated cells in G_0_/G_1_ that have arisen after one endocycle. Because we found no evidence for increased cell division in *exi* leaves, the enlarged 2C and 4C populations observed in the *exi* mutants are likely derived from the small proportion of cells that enter the endocycle in these mutants. Our results indicate that cell size and endoreduplication levels are positively correlated in the *exi* leaves, suggesting that the function of the *EXI* genes in the regulation of cell expansion is coupled with endoreduplication levels during leaf growth.

**Table 1:** Nuclear DNA ploidy distribution in the first pair of leaves of the *exi* mutants.

Genotype	Ploidy level				
	2C	4C	8C	16C	32C
L*er*	23.4±2.8 (22.3)	27.5±1.1 (27.7)	35.0±1.3 (35.2)	13.3±2.6 (13.9)	0.8±0.3 (0.7)
*exi1-2*	***29.2±1.2 (29.1)***	27.3±1.1 (27.4)	**30.8±2.3 (30.4)**	11.2±2.0 (12.4)	1.5±0.6 (1.6)
*exi2*	***27.6±1.0 (27.5)***	***34.5±1.1 (34.3)***	**31.9±1.5 (31.9)**	**5.4±0.8 (5.4)**	0.6±0.1 (0.7)
*exi5*	***32.7±1.3 (32.7)***	***34.1±0.6 (34.1)***	**28.8±1.2 (28.9)**	**3.9±0.5 (4.0)**	0.5±0.1 (0.5)

Values are mean±standard deviation. The median is shown in parentheses. Significant differences (*P* value<0.01, n = 10) from the L*er* control are in bold. Values higher than those of L*er* control are in italics. Data analysis was performed as indicated in Materials and Methods. All plants were homozygous for the mutations indicated.

### The *EXI* Genes Encode the Three Cellulose Synthase Subunits

**Figure 4:**
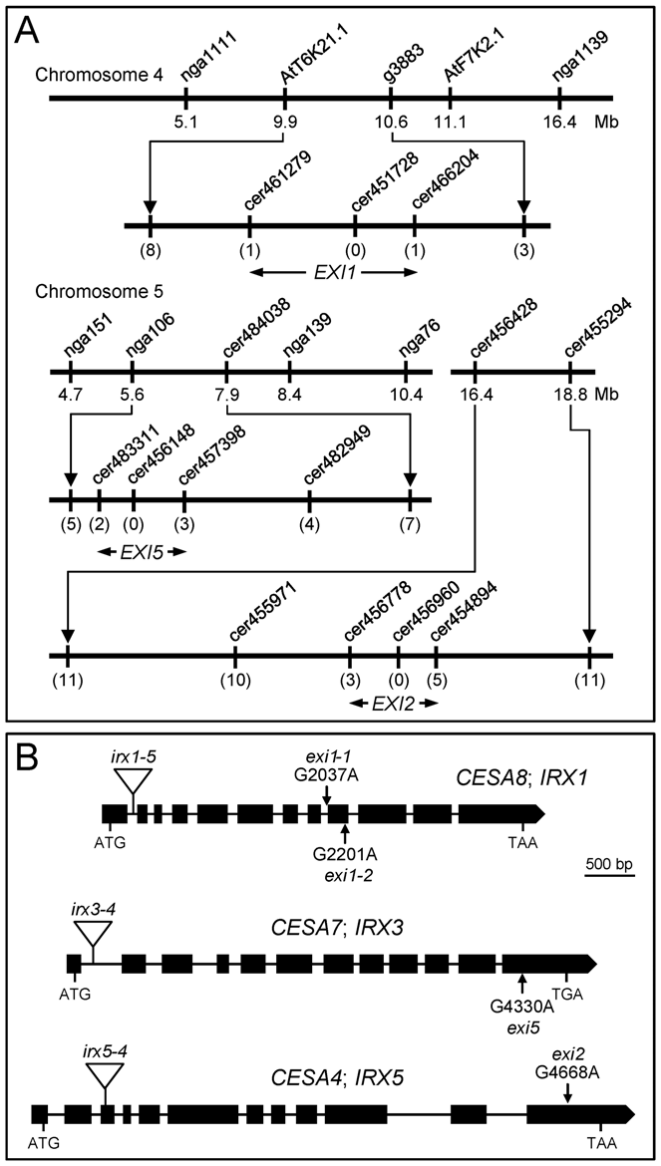
Positional cloning of the *EXI1*, *EXI2* and *EXI5* genes. (A) Map-based cloning strategy used to identify the *EXI* genes. Mapping populations of 217 F_2_ plants were derived from an *exi1-1*×Col-0 cross, 417 from an *exi2*×Col-0 cross, and 278 from an *exi5*×Col-0 cross. The candidate intervals for *EXI1*, *EXI2* and *EXI5* were of 231, 233 and 390 kb, respectively, which encompassed 70, 55 and 110 annotated genes. The molecular markers used for linkage analysis, their map positions and the number of informative recombinants identified (in parentheses) are indicated. (B) Structure of the *EXI1*, *EXI2* and *EXI5* genes, with indication of the nucleotide position (relative to the translation start site) and nature of the mutations found; a triangle indicates a T-DNA insertion. Exons are represented as boxes, and introns by lines connecting boxes. The translation start (ATG) and stop (TAA or TGA) codons are also indicated.

Because none of the *exi* mutations under study was tagged, we followed a positional cloning approach for their molecular identification. Fine mapping of the *exi1-1*, *exi2* and *exi5* mutations defined candidate intervals encompassing 70, 55 and 110 genes, respectively (Figure 4A). The four *exi* mutants studied shared a similar leaf phenotype (see above), and we reasoned that the *EXI* genes might be functionally related. Indeed, each of the three genomic intervals found contained a gene encoding a catalytic subunit of the cellulose synthase A complex: *CESA8* (also named *IRX1*; At4g18780) [19], *CESA7* (*IRX3*; At5g17420) [20] and *CESA4* (*IRX5*; At5g44030) [15] were included within the *exi1-1*, *exi5* and *exi2* candidate intervals, respectively. Interestingly, the Arabidopsis genome encodes 10 CESA proteins [21], of which only those produced by the *CESA4*, *CESA7*, and *CESA8* genes are integrated into the CESA complex that is responsible for the biosynthesis of secondary cell walls [22]. The *exi1-1* mutant was found to carry a G→A transition in the acceptor site of the 8^th^ intron of the *CESA8* gene (Figure 4B), thus altering its mRNA splicing (Figure S2). The *exi1-2* mutation (Figure 4B) changes a conserved glycine residue to glutamic acid in the second cytoplasmic domain of the CESA8 protein that is required for substrate binding and catalysis [23]. The *exi5* mutation (Figure 4B) results in the introduction of a stop codon in place of the tryptophan 954 residue, which causes a premature termination of translation. The *exi2* mutation affects the last exon of the *CESA4* gene (Figure 4B) and introduces a premature stop codon that removes 109 residues from the CESA4 C-terminus.

Homozygotes for a T-DNA insertion in the *CESA8* gene were obtained from the Salk_026812 line [24], which exhibited the same phenotype already observed in the *exi* mutants (we will hereafter refer to this allele as *irx1-5*; Figure S3). We confirmed the presence of the T-DNA insertion in the first intron of *CESA8* in the *irx1-5* plants (Figure 4B). The F1 progeny of an *irx1-5* × *exi1-1* cross were phenotypically similar to the *exi1-1* parental, confirming that *irx1-5* and *exi1-1* are allelic. Rosettes of the Salk_029940C line [24], [25], which bear a T-DNA insertion in the first intron of *CESA7* (*irx3-4* in Figure 4B), also display a small-leaf phenotype (Figure S3), indicating allelism with our *exi5* mutants. Complementation tests also allowed us to demonstrate allelism between *exi2* and the T-DNA insertion in the third exon of *CESA4* (*irx5-4* in the Figure 4B), carried by the Salk_084627 line [24], [25], which causes small, dark-green vegetative leaves (Figure S3).

### The *Exi* Mutants Show Altered Vascular Tissue Morphology

**Figure 5:**
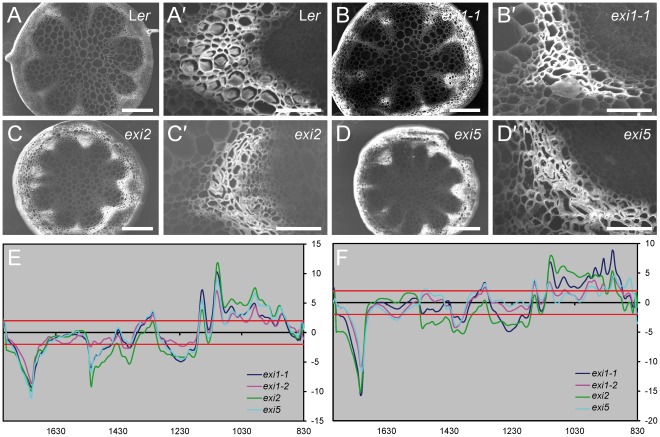
Xylem structure and composition in *exi* stems. (A–D) Scanning electron micrographs of transverse sections of stems from (A, A′) L*er*, (B, B′) *exi1-2*, (C, C′) *exi2* and (D, D′) *exi5* plants. Magnification is (A–C) 70x and (A’–C’) 500x. (E–F) Comparison of the Fourier transform infrared spectroscopy spectra obtained from stem sections of L*er* and *exi* plants. Spectra were acquired on xylem in apical (E) and basal (F) parts of the stem. The value between the two red lines (threshold) corresponds to non-significant differences (*P* value<0.01) between the two genotypes tested. Significant positive *t*-values indicated a higher absorbance value in L*er* than in the *exi* mutants.

**Figure 6:**
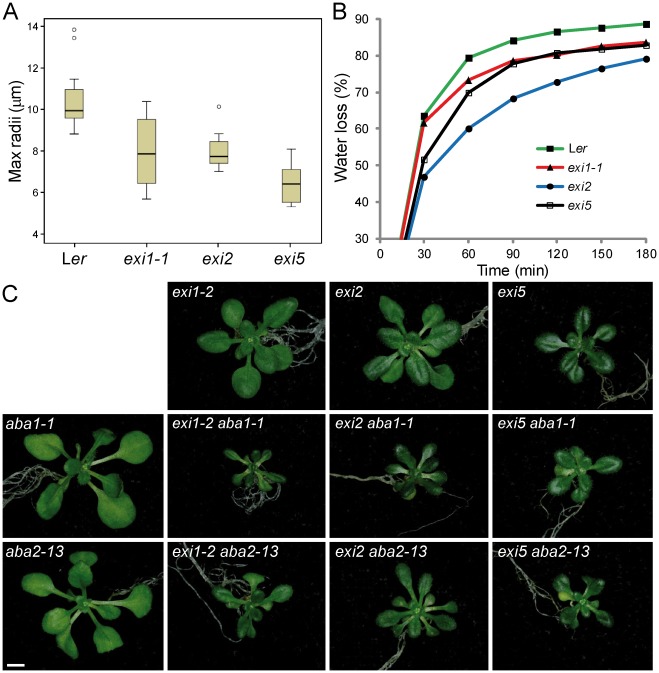
Water loss and xylem vessel size in the *exi* mutants. (A) Box plots showing maximal xylem vessel radius data for the *exi* mutants. Each box plot was produced from 15 measurements. All differences between each mutant and the wild type were statistically significant (*P* value<0.05). Outliers are shown as circles. (B) Rapid dehydration of detached rosettes from 4 week-old plants. Water loss was determined as percentage of initial fresh weight. (C) Rosette phenotypes caused by the *exi*, *aba1-1* and *aba2-13* mutations and their double mutant combinations. Pictures were taken 21 DAS. The scale bar indicates 1 mm.

Mutations in genes encoding CESA complex components cause an irregular xylem phenotype: xylem vessels are collapsed as a consequence of reduced cellulose deposition in their cell walls [26]. To analyze whether our *exi* mutants display similar vascular defects, we performed scanning electron microscopy on transverse sections of inflorescence stems (see Materials and Methods). As expected from the molecular function of the *EXI* genes in secondary cell wall biosynthesis, all the *exi* mutants that we studied displayed collapsed xylem vessels in the vascular bundles of the inflorescence stem, whereas their parenchyma cells were of normal morphology but of smaller sizes than those of L*er* (Figure 5A–D). Based on these observations, we hypothesized that hydraulic conductivity might be altered in the *exi* mutants. According to Poiseuillés law, theoretical hydraulic conductivity of a vessel is proportional to the fourth power of its radius [27], which implies that little differences in the vessel radius can lead to great changes in hydraulic conductivity. We measured the maximal radii from 15 xylem vessels per genotype (Figure 6A), finding that they were significantly smaller in the *exi* mutants than in L*er*.

The chemical composition of the vascular tissues from the inflorescence stems was examined using Fourier transform infrared (FTIR) spectral analysis. A Student’s *t*-test was performed to evaluate the differences between the L*er* and *exi* spectra. For graphical representation, the *t*-values were plotted against wavenumbers to determine the significance of the differences at each wavenumber [28]. Our results suggest that all three mutant classes had very similar cell wall changes in the vascular tissue of the stem in the three *exi* mutants analyzed (Figure 5E–F), including a reduced cellulose content as suggested by the positive t value at the wavenumbers 1319, 1157, 1106, 1060, 1037 and 990 cm^-1^, correlated with an increase of lignin content as suggested by the negative t value at the wavenumbers 1708 and, 1515 to 1392 cm^-1^ [28]. Defective secondary cell wall biosynthesis in the xylem vessels of *exi* stems, caused by impaired cellulose biosynthesis, might increase the sensitivity of the xylem to a high negative water pressure, which, in turn, would cause the observed collapse of the xylem.

### The *Exi* Mutants Display Increased Resistance to Osmotic Stress

A number of physiological processes, such as osmotic stress responses, are regulated by water availability. Because the *exi* mutants show collapsed xylem vessels, water transport might be obstructed in these mutants and, as a consequence, the *exi* mutants might be affected in their osmotic stress responses.

**Figure 7:**
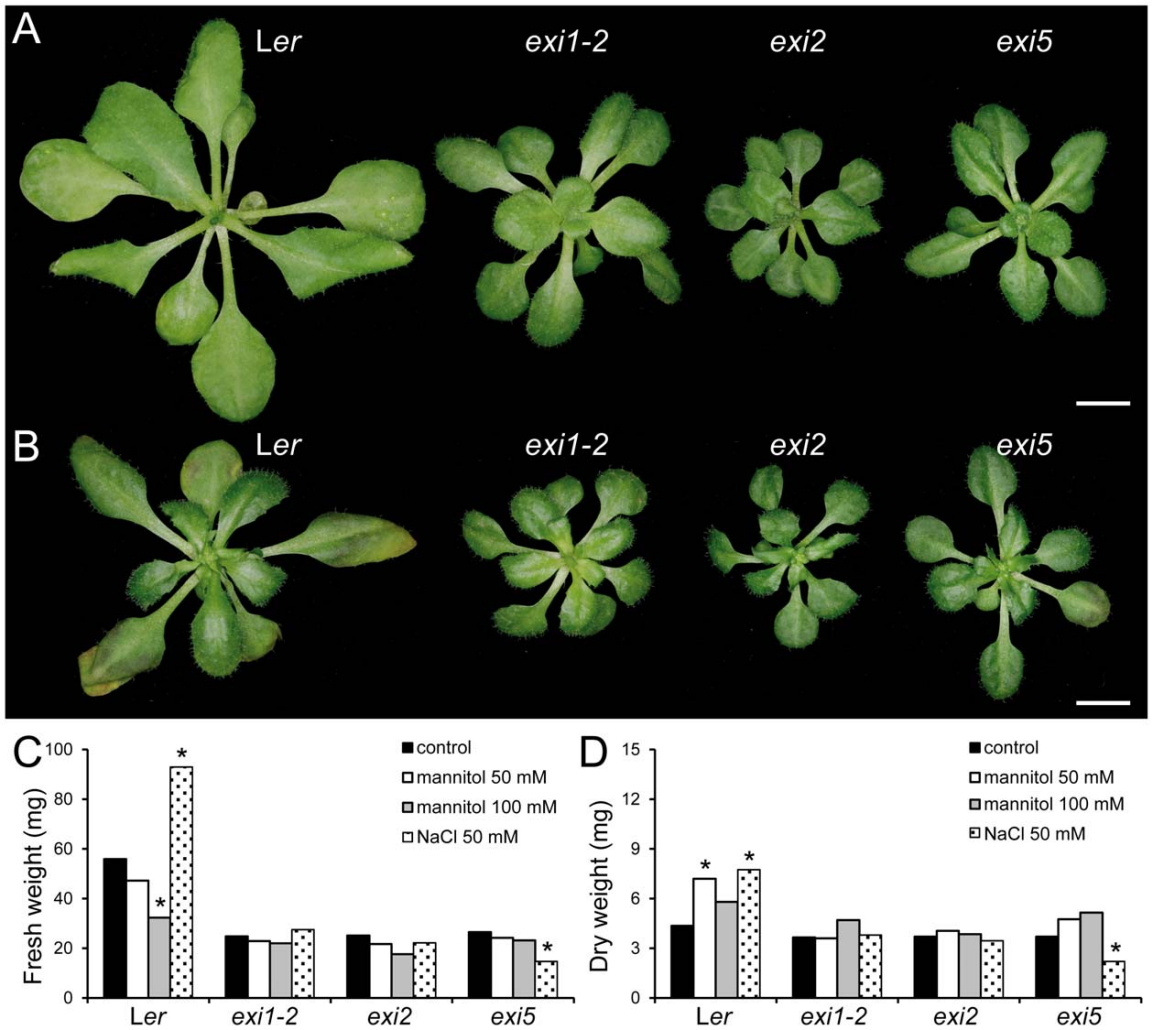
Osmotic stress tolerance of the *exi* mutants. (A–B) Rosette morphology in seedlings grown on media (A) without mannitol or (B) supplemented with 100 mM mannitol (B). (C, D) Effects of different concentrations of mannitol or NaCl on the (C) fresh weight (D) dry weight of 26-day-old rosettes. Plants were grown for 19 days on half-strength MS agar medium and then transferred to half-strength MS agar plates supplemented or not with either mannitol or NaCl at different concentrations. Photographs were taken after one week of treatment, and rosettes were immediately weighed (fresh weight) or dried for 16 h at 60°C and then weighted (dry weight). Asterisks indicate significant differences from the control plates without mannitol or NaCl (*P* value<0.01). Each column represents the mean of three measurements (8–10 rosettes each). Scale bars indicate 2 mm.

We first tested the responses of *exi* mutants to rapid dehydration (see Materials and Methods). Consistent with the hypothesis that the morphological alterations in xylem vessels impair water transport in the *exi* mutants, their dehydration rates were reduced compared to L*er* (Figure 6B). We assayed the osmotic stress responses by transferring young plants to media supplemented with different concentrations of the osmoticum mannitol or NaCl and by measuring the rosette fresh weight and dry weight after one week of growth on either of these media (see Materials and Methods). The rosette fresh weight (Figure 7C) but not the dry weight (Figure 7D) was significantly decreased in L*er* plants grown on mannitol, due to the severe water loss on the osmoticum-supplemented media. Similarly, the rosette fresh weight was greatly reduced in the abscisic acid (ABA)-deficient mutant *aba2-13* [29] when grown on mannitol-supplemented plates, even at low levels (50 mM mannitol). The *exi* mutants, however, were more resistant to mannitol-induced water losses, and their rosette fresh weights were not significantly affected by increasing the level of mannitol (Figure 7C). The rosettes of L*er* plants grown for one week on 100 mM mannitol displayed a severe growth reduction and enhanced senescence in older leaves, whereas the *exi* mutant rosettes showed less obvious morphological alterations on high mannitol concentrations (Figure 7A–B). The L*er* rosettes grown on media supplemented with 50 mM NaCl displayed increased fresh weights after one week due to their higher water content, compared with the plants grown on the control media (Figure 7C–D). In contrast, the fresh weights of the *exi* rosettes grown for one week on media containing 50 mM NaCl were not significantly different from those of the *exi* plants grown on the non-supplemented media (Figure 7C–D), which clearly indicates a defect in water transport in the *exi* mutants. Taken together, our results suggest that the increased resistance to osmotic stress and the low responses of *exi* mutants to moderate NaCl levels are caused by defective water flux regulation due to xylem collapse in the *exi* mutants.

### The *EXI* Genes Act Independently from the ABA Biosynthetic Pathway

ABA is involved in many environmental stress responses, particularly cold, salinity and drought [30]. In response to drought, an increase in the endogenous ABA levels limits transpirational water loss through the regulation of stomatal closure. ABA-deficient mutants, like *aba1* and *aba2*, display a stunted (wilty) phenotype, even under well-watered conditions, as a consequence of their inability to retain water because of impaired stomatal closure [31], [32]. Based on the higher ABA levels observed in the *cesA8* mutant, a crosstalk between secondary cell wall integrity and ABA signaling pathway has been proposed [33], [34]. To test whether ABA accumulation in the *exi* mutants might be, at least partly, responsible for the Exi phenotype, we generated double mutant combinations between our *exi* mutants and either the *aba1-1* or *aba2-13* ABA-deficient mutants (Figure 6C). The *aba1-1* and *aba2-13* single mutants exhibit a wilty phenotype and their rosettes are slightly smaller than those of L*er* [31], [32]. The rosette phenotypes of the six *exi aba* double mutant combinations studied were all similar: they displayed small and darker-green vegetative leaves as in their *exi* parental, as well as the wilty phenotype characteristic of their ABA-deficient parental (Figure 6C and data not shown). In addition, rosette size was more reduced in the double mutants than in any of their parentals. We interpreted the phenotype of the *exi aba* double mutants as additive, an observation that also suggests that the phenotype of the *exi* mutants is not directly caused by high levels of ABA.

### Cell Expansion Defects of the *Exi* Mutants are Rescued by Liquid Culture

The cellulose biosynthesis activity encoded by the three *EXI* genes studied here is restricted to the deposition of secondary walls of developing xylem [35]. In the *exi* mutants, however, all the leaf cells –including those without secondary walls– display small sizes, despite the fact that most of them lack secondary cell walls. We then addressed the possible causes of the observed non-cell autonomy of this phenotype. It has been suggested that water uptake into the vacuole creates the internal turgor pressure necessary for the extension of the cell wall through the synthesis of new wall components [36]. Because cells in the *exi* leaves are impaired in water uptake, we reasoned that the internal turgor pressure required for primary cell wall extension during leaf growth is limited in the *exi* mutants. Therefore, the primary cell wall is not directly affected by the *exi* mutations, and experimentally increasing the turgor pressure in the *exi* leaves should rescue their small-cell phenotype. Alternatively, changes in the secondary cell wall composition in the *exi* mutants might be sensed by younger growing tissues, leading to a complex alteration in the composition of the primary cell wall in newly formed organs, as has previously been proposed [37].

**Figure 8:**
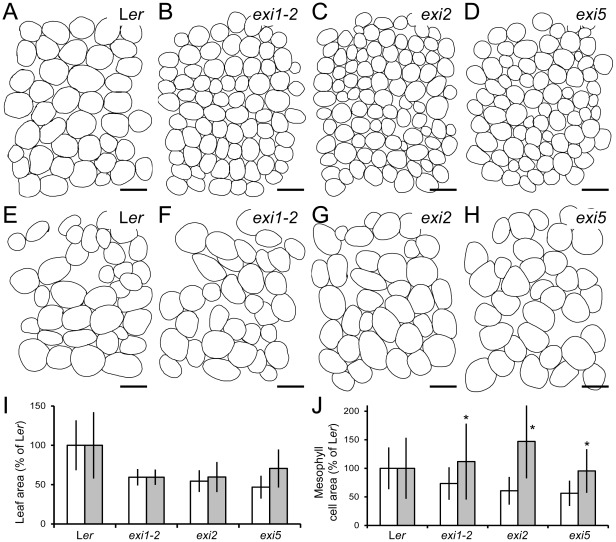
The cell expansion defects of the *exi* mutants are rescued by liquid culture. (A–H) Leaf mesophyll of the *exi* mutants. Diagrams were drawn from differential interference contrast micrographs of cleared first-node leaves collected from (A, E) L*er*, (B, F) *exi1-2*, (C, G) *exi2* and (D, H) *exi5* plants. (I, J) First-node leaf (I) lamina area and (J) mesophyll cell area in the *exi* mutants. Data were normalized with respect to the L*er* values and are expressed as percentages (mean and standard deviation). Asterisks indicate significant differences from the control seedlings grown on half-strength MS agar medium (*P* value<0.01). All the data were obtained 21 DAS from seedlings grown on half-strength MS agar plates (A–D, white bars) or in half-strength MS liquid cultures (E–H, grey bars). Scale bars indicate 50 µm.

To ensure that water uptake was independent from its transport through the vascular tissue, L*er* and the *exi* mutants were grown for three weeks in 10 ml of half-strength MS liquid medium (see Materials and Methods). In this situation, water freely enters through the stomata into the leaf mesophyll, where the increase in internal turgor pressure might trigger cell expansion. Although *exi* leaves grown in liquid culture remained smaller than those of L*er* (Figure 8I), the size of the palisade mesophyll cells in the L*er* and *exi* leaves grown in liquid culture did not significantly differ (Figure 8J). Indeed, the palisade mesophyll cells of the *exi* mutants grown in liquid culture were of similar sizes as the L*er* mesophyll cells grown on Petri dishes (Figure 8A–H). Our results confirm the rescue of primary cell wall expansion in the palisade mesophyll cells of the *exi* mutants by experimentally increasing the water uptake in these cells. Thus, the small cells of the *exi* mutant leaves are caused by impaired water transport through the xylem vessels due to their collapse caused by defective secondary cell wall formation.

## Discussion

Forward genetic screening in Arabidopsis is a powerful tool that has helped to identify several key loci involved in leaf development [8], [38], [39]. The final leaf size relies on the coordination between cell proliferation and post-mitotic cell expansion [1]. To dissect the regulation of cell expansion genetically, we phenotypically and molecularly characterized four *exigua* (*exi*) mutants that show decreased cell size but normal cell numbers in their leaves.

We found that the *EXI* genes encode three subunits of an essential cellulose synthase (CESA) complex required for secondary cell wall biosynthesis: CESA4 (EXI2), CESA7 (EXI5), and CESA8 (EXI1) [15]. In xylem vessels, these three subunits co-localize with the sites of secondary cell wall deposition. Based on the molecular damage observed in *exi1-1* and *exi1-2*, both alleles are likely hypomorphic and do not completely abolish the CESA function. In line with this hypothesis, the *exi1* mutations confer the mildest phenotype among the *exi* mutants studied. In contrast, the *exi5* mutation presumably causes a strong loss of function because it produces a truncated CESA7 protein. *irx3-4* plants, which are homozygous for a T-DNA insertion in the first intron of *CESA7* that abolishes its expression [25] display a mutant phenotype that is indistinguishable from that of *exi5*. The *exi2* missense mutation produces a truncated CESA4 protein causing a phenotype that is also indistinguishable from that associated to the *irx5-4* T-DNA insertional allele of *CESA4* (this work). Interestingly, the *exi2 exi5* double mutant phenotype is stronger than either of the parental phenotypes, suggesting that the CESA complex can still function in the absence of one of its subunits, likely due to redundancy among CESA subunits. Consistent with our hypothesis, some degree of functional redundancy between the protein subunits of the CESA complex implicated in primary cell wall biosynthesis has previously been described [40], [41]. Alternatively, if the truncated CESA subunits of the *exi2* or *exi5* mutants integrate into the complex, the stronger phenotype observed in the *exi2 exi5* double mutants might be caused by a dominant negative effect of the mutant alleles that leads to a partial (and cumulative) reduction in the activity of the CESA protein complex. Protein purification and immunolocalization of the CESA complexes in our *exi* mutants will help us to distinguish between these possibilities.

Plant cell walls are complex structures that are involved in key processes, such as cell elongation, drought responses and plant-pathogen interactions [42]. Recent work has provided evidence for a functional connection between those apparently distinct processes and cell wall integrity [43]. In fact, the disruption of *CESA8* (*EXI1*) resulted in an enhanced tolerance to drought and osmotic stresses [33]. Interestingly, the *cesA8* mutants contain higher levels of abscisic acid (ABA) and demonstrate an enhanced expression of the genes encoding key components of the ABA biosynthetic and signaling pathways [33], [34]. Plants respond to soil drought by closing their stomata to reduce water loss, a process that is regulated by ABA [44]. Moreover, dehydration of excised pine needles causes the reversible collapse of xylem vessels [45] in a pattern that resembles that of the *exi* mutants studied here. The enhanced resistance of *cesA8* [33] and our *exi* mutants (this work) to osmotic stresses might be caused by enhanced ABA levels or responses that, in turn, trigger stomata closure to reduce water losses through transpiration and maintain a low water potential that prevents dehydration [46], [47]. Alternatively, collapsed xylem vessels in the *cesA8* and *exi* mutants directly reduce the osmotic stress-induced water losses due to defective water transport through the xylem. Our results with the *exi aba* double mutants indicate that ABA biosynthesis and secondary cell wall biosynthesis function independently for the regulation of osmotic stress tolerance, the first by the regulation of stomatal closure and the second by the regulation of turgor pressure.

Cell expansion is driven by the internal turgor pressure created by osmotic water uptake while is restricted by the extensibility of the cell wall, a process that is regulated by the synthesis, incorporation, and cross-linking of new cell wall components [36], [48]. Inhibition of cellulose biosynthesis, either genetically or chemically, leads to the activation of an unknown mechanism that monitors cell wall integrity and prevents further growth [49], [50]. The molecular function of the *EXI* genes provides a causal link for their mutant phenotype of reduced cellulose biosynthesis in secondary cell walls (i.e., xylem vessels). However, cells without secondary walls, such as those in the palisade mesophyll of *exi* leaves, also show defective cell expansion in these mutants. One possibility is that secondary cell wall changes are sensed by the above-mentioned mechanisms to affect downstream signaling events, leading to local and systemic alterations in primary cell wall composition in the *exi* mutants. Our results, however, are consistent with the alternative hypothesis that the collapsed xylem vessels in the *exi* mutants cause reduced water transport throughout the plant and compromise the turgor pressure required during cell expansion. By experimentally increasing the turgor pressure in the palisade mesophyll cells of *exi* leaves, we were able to fully rescue their cell expansion defects, demonstrating that primary cell wall biosynthesis is not directly affected by the *exi* mutations. We postulate that the observed tolerance of the *exi* mutants to osmotic stresses is also an indirect consequence of impaired water transport due to their collapsed xylem vessels. Moreover, hydraulic conductivity through the vessels is expected to be compromised in the *exi* mutants. Consistent with our hypothesis, mutations in the *ESKIMO1* gene that encodes a member of a family comprising xyloglucan actyltransferases [51], which is expressed in tissues undergoing secondary cell wall deposition [52], [53], also produce a collapsed xylem phenotype and a concomitant reduction in the water supply to the vegetative tissues [52]. Similar to that found in the *eskimo1* mutants [52], the *exi* mutants exhibit low theoretical hydraulic conductivity and a likely diminished *in planta* hydraulic conductance due to restricted water transport through their collapsed vessels.

Strong correlations between the ploidy level and cell size have been reported in many plant species. Although the transition from cell proliferation to cell expansion is often accompanied by the transition from the mitotic cycle to the endocycle [18], some evidence suggests that endoreduplication and cell expansion could be uncoupled [54]. A recent example is provided by the constitutive expression of the basic helix-loop-helix transcription factor ROOT HAIR DEFECTIVE 6-LIKE 4 (RSL4), which leads to continuous growth of hair cells without an increase in the DNA ploidy content [55]. However, a decrease in cell proliferation in Arabidopsis leaf primordia below some threshold level triggers enhanced post-mitotic cell expansion [10], such as that caused by the overexpression of the cyclin-dependent kinase (CDK) inhibitor gene *KIP-RELATED PROTEIN2* (*KRP2*) [56]. We found that the *exi* leaves show a global reduction of endoreduplication levels (>4C) and a slight increase in the size of their diploid cell population. Our results indicate that in the *exi* mutants, as occurs in wild-type Arabidopsis leaves [17], cell size and ploidy levels are positively correlated, suggesting crosstalk between the cell wall integrity and regulation of the endocycle. The WEE1 kinase is a conserved negative regulator of CDK activity during the G2 phase of the cell cycle [57], and WEE1 orthologs in yeast have been linked to cell size control [58]. The misexpression of the WEE1 kinase in tomato resulted in dwarf plants due to a reduction in cell size that correlated with a decrease in their DNA ploidy levels [59]. It will be interesting to test whether the activity of some of the regulators of endoreduplication, such as WEE1, is dependent on changes in the internal turgor pressure.

The results of our studies with the *exi* mutants support the hypothesis that the compensated cell enlargement mechanism observed in Arabidopsis leaves operates mainly by monitoring the extent of cell proliferation. We hypothesize that an increase in turgor pressure precedes the new cell wall biosynthesis that triggers enhanced cell expansion in post-mitotic cells during compensation. One candidate to be involved in regulating turgor pressure is vacuolar H^+^-ATPase (V-ATPase), which is responsible for proton translocation from the cytosol. The V-ATPase function is impaired in the *de-etiolated3* (*det3*) mutant, which displays a severe defect in normal cell expansion [60]. The light-grown *det3* phenotype is due to hormone-mediated changes in gene expression that, in turn, prohibit cellulose biosynthesis [61].

Our detailed phenotypic analysis of the leaves of the *exi* mutant suggests a mechanism for cell expansion-mediated organ growth that acts through the regulation of water transport and that monitors internal turgor pressure. Our *exi* mutants will serve as molecular tools to dissect the complex relationship between cell wall integrity and cell cycle regulation during organ growth.

## Materials and Methods

### Plant Material and Growth Conditions

*Arabidopsis thaliana* (L.) Heynh. *exi1-1*, *exi1-2*, *exi2* and *exi5* mutants were isolated as described in [5]. Salk_026812 (*irx1-5*), Salk_029940 (*irx3-4*) and SalK_084627 (*irx5-4*) T-DNA insertion lines were obtained from the Nottingham *Arabidopsis* Stock Centre (NASC; http://arabidopsis.info/) and are described at the SIGnAL website [24] (http://signal.salk.edu). We confirmed the T-DNA insertion sites of the mutant lines by PCR amplification and/or sequencing.

Sterile plant cultures were performed as described in [62], on 150-mm diameter Petri dishes containing 100 ml of half-strength Murashige and Skoog agar medium supplemented with 1% sucrose (MS medium). The plants were grown at a density of 50 regularly spaced seedlings per plate. Sterile cultures were incubated in Conviron TC16 growth chambers (Conviron, Winnipeg, Canada; http://www.conviron.com/) at 20 ± 1°C and 60–70% relative humidity, and with continuous illumination of 78 µmol m^-2^ s^-1^.

### Morphological and Ultrastructural Analyses

Images of rosettes and leaves were taken 21 days after stratification (DAS) with a Panasonic DMC-FX9 digital camera (2,816×2,112 pixels; Osaka, Japan). For quantification of the cell areas, diagrams of the adaxial epidermis and the underlying palisade mesophyll from cleared leaves [63] were obtained by drawing the outlines of the cells on a Wacom Cintiq 18SX Interactive Pen Display (Wacom Company Ltd., Tokyo, Japan; http://www.wacom.com/). The cell areas were measured from these images using the NIS Elements AR 2.30 image analysis package (Nikon, Tokyo, Japan; http://www.nis-elements.com/index.html) as described in [64]. Photomicrography and confocal microscopy of whole leaves and leaf sections were performed as previously described elsewhere [65], [66]. All the data were statistically analyzed with SPSS (Statistical Package for the Social Sciences) 15.0 software (SPSS Inc., Chicago, IL, USA) [67] and graphically represented and digitally processed with Adobe Photoshop and Illustrator CS3 (Adobe Systems Incorporated, San Jose, CA, USA; http://www.adobe.com/). For dehydration assays, the entire root system was excised from a minimum of four 21 DAS rosettes per genotype and water loss was measured as previously described [52]. In micrographs taken from stem transverse sections, we measured the maximal radii of all vessels in 5 to 10 xylem poles per genotype, using the ImageJ software (http://rsb.info.nih.gov/ij/). The data from the 15 larger xylem vessels of each genotype were statistically analyzed with SPSS.

### Ploidy Analysis

Flow-cytometry analysis was performed as previously described [68]. Briefly, first-node and second-node leaves from 4 different rosettes were harvested 21 DAS and chopped with a razor blade in 500 µl of cold nuclear isolation buffer [69]. The cell suspension was then filtered over 30-µm nylon mesh, treated with RNase A (200 µg ml^-1^) and stained with propidium iodide (50 µg ml^-1^) for 10 min. The nuclear DNA content was then analyzed using a FACS Canto II flow cytometer (BD Biosciences, Franklin Lakes, NJ, USA; http://www.bdbiosciences.com/), and the data were processed using FACSDiva 6.1.2 software. A minimum of three biological replicates, including 10,000 counts each, were studied per genotype.

### Positional Cloning and Sequencing of *EXI* Genes

To positionally clone the *EXI1*, *EXI2* and *EXI5* genes, a low-resolution map was constructed according to [70], [71]; for fine mapping, SSLP, SNP and InDel markers were designed based on the polymorphisms between the L*er* and Col-0 ecotypes described in the Arabidopsis Polymorphism Database (http://www.arabidopsis.org). For sequencing of the alleles, PCR products spanning the At4g18780, At5g17420 and At5g44030 transcriptional units were obtained using wild-type and mutant genomic DNA as the templates and the oligonucleotide primers shown in Table S1. The sequencing reactions were carried out with the ABI PRISM BigDye Terminator cycle sequencing kit (Life Technologies Corporation, Carlsbad, CA, USA; http://www.appliedbiosystems.com) in 5-µl reaction volumes. Sequencing electrophoreses were performed with an ABI PRISM 3130xl Genetic Analyzer (Applied Biosystems).

### RT-PCR Analysis

Total RNA was extracted from 3-week-old wild-type and *exi1-1* rosettes using TRIzol (Life Technologies Corporation, Carlsbad, CA, USA; http://www.invitrogen.com/), as instructed by the manufacturer. A 2-µg aliquot of the mRNA was heated for 5 min at 65°C and immediately transferred to ice. 500 ng of random hexanucleotides (Roche Applied Science, Basel, Switzerland; http://www.roche-applied-science.com/) were added and the mixture was then incubated at room temperature for 10 min. First-strand cDNA was synthesized in a 20-µl reaction mixture containing 0.5 mM of each deoxyribonucleoside triphosphate, 10 mM dithiothreitol, 200 units of SuperScript II enzyme (Life Technologies Corporation), and 40 units of RNaseOUT. The primer pairs used (EXI1_3bF and EXI1_3R) are shown in Table S1.

### Scanning Electron Microscopy

Segments (1 cm) of Arabidopsis inflorescence stems were embedded in an 8% agarose solution in sterilized/RNAse-free water at 50°C. After cooling, the blocks were sectioned using a vibratome. The 100-µm sections were observed immediately after sectioning using a Hirox SH-1500 scanning electron microscope (Hirox Co. Ltd., Tokyo, Japan; http://www.hirox.com/).

### Fourier Transform Infrared (FTIR) Spectroscopy

Analyses were carried out on the xylem and parenchyma tissue from the basal and upper regions of branched stems using 50-µm-thick vibratome sections of agarose-embedded tissue. For each genotype, five to six sections from three different plants were analyzed. The FTIR spectra were collected from a window (50 µm x 50 µm) targeting the xylem vessels using the IN10 FTIR imaging system (Thermo Fisher Scientific, Waltham, MA, USA; http://www.thermoscientific.com/); normalization of the data and statistical analysis (Student’s *t*-test) were performed as described in [72].

### Physiological Analyses and Liquid Culture

For the osmotic stress analyses, sterile cultures were performed as described above. The plants were grown for 19 days on half-strength MS agar plates, and a minimum of eight plants per genotype were transferred to half-strength MS agar plates that had either not been supplemented or supplemented with mannitol (50 mM or 100 mM) or NaCl (50 mM). After one week of treatment, photographs were taken and the rosettes were immediately collected and weighed (fresh weight), or dried for 16 h at 60°C and then weighed (dry weight). All the genotypes were sown on triplicate plates.

For the turgor-mediated cell-size rescue of the *exi* mutants, the plants were grown for 21 days in 50-ml Falcon conical tubes containing 10 ml half-strength MS liquid medium at 20 ± 1°C and 60–70% relative humidity, with continuous illumination (78 µmol m^-2^ s^-1^) and continuous shaking (100 rpm). The morphometric analyses of the cell area of the adaxial epidermal and palisade mesophyll cells were performed as described above.

## Supporting Information

Figure S1 **Additive phenotype of the** ***exi2 exi5*** **double mutant.** Pictures of (A) *exi2* and (B) *exi2 exi5* were taken 27 DAS. Scale bars indicate 3 mm.
(TIF)

Figure S2 Agarose gel electrophoresis of RT-PCR amplification products obtained from *CESA8* transcripts. RNA was extracted from *exi1-1* (lanes 1, 2) and L*er* (lanes 3, 4) rosettes collected 21 DAS, reverse transcribed and PCR amplified using the EXI1_3bF and EXI1_3R primers (Table S1). Lane 5: 1 kb DNA ladder (Invitrogen).
(TIF)

Figure S3 **Mutants carrying T-DNA insertional alleles of the** ***EXI*** **genes.** Rosettes are shown from (A) Col-0, (B) *irx1–5* (Salk_026812), (C) *irx5-4* (Salk_084627) and (D) *irx3–4* (Salk_029940). Pictures were taken 21 DAS. Scale bars indicate 2 mm.
(TIF)

Table S1 **Primers used in this work.**
(DOCX)
