# Supplementary figures and images for: Cell Expansion-Mediated Organ Growth Is Affected by Mutations in Three EXIGUA Genes
Source: PLoS One. 2012 May 4;7(5):e36500. doi: 10.1371/journal.pone.0036500 (PMC3344895; doi:10.1371/journal.pone.0036500)

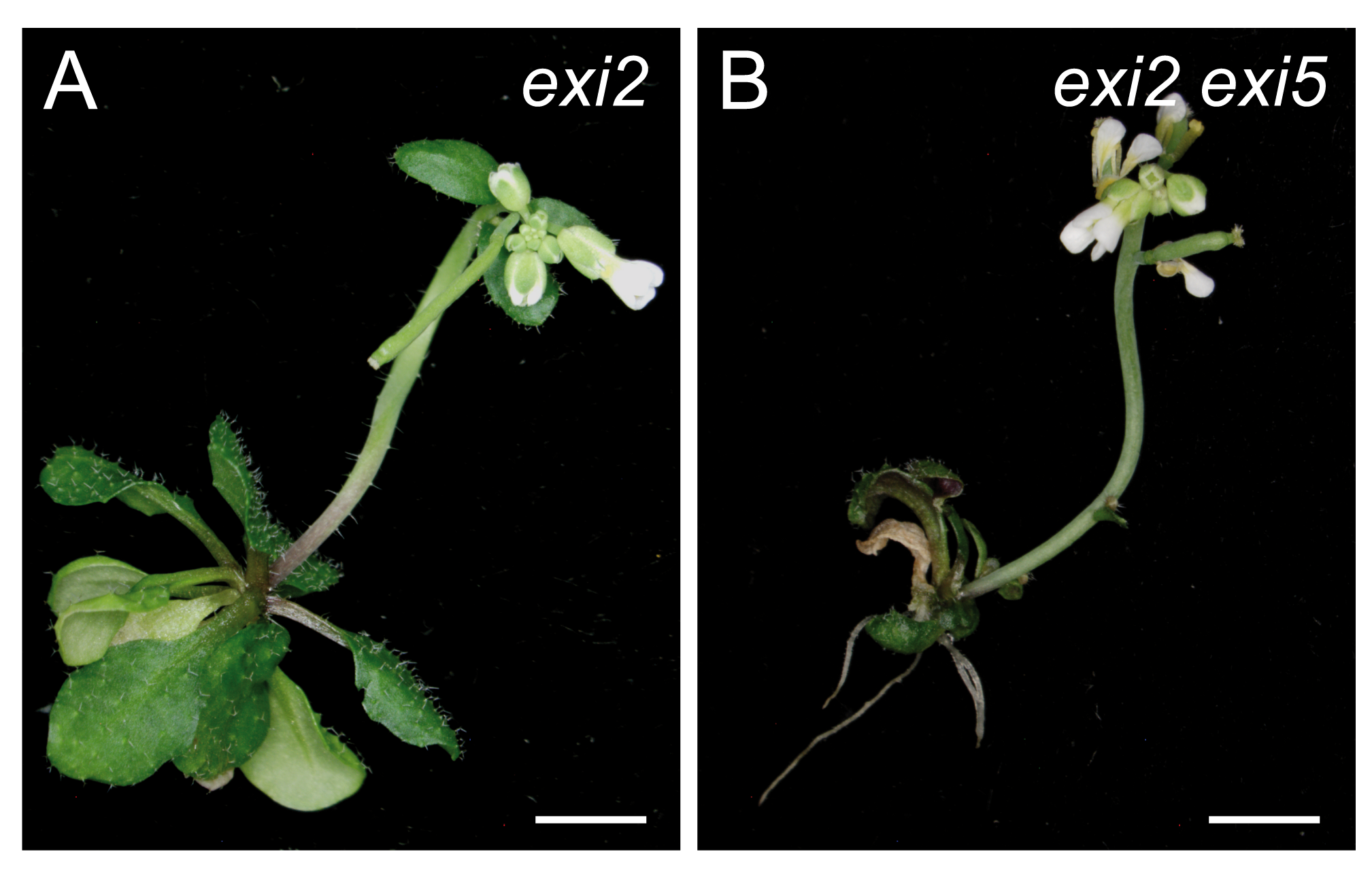

Supplement: Figure S1 — Additive phenotype of the exi2 exi5 double mutant. Pictures of (A) exi2 and (B) exi2 exi5 were taken 27 DAS. Scale bars indicate 3 mm. (TIF) [file pone.0036500.s001.tif]

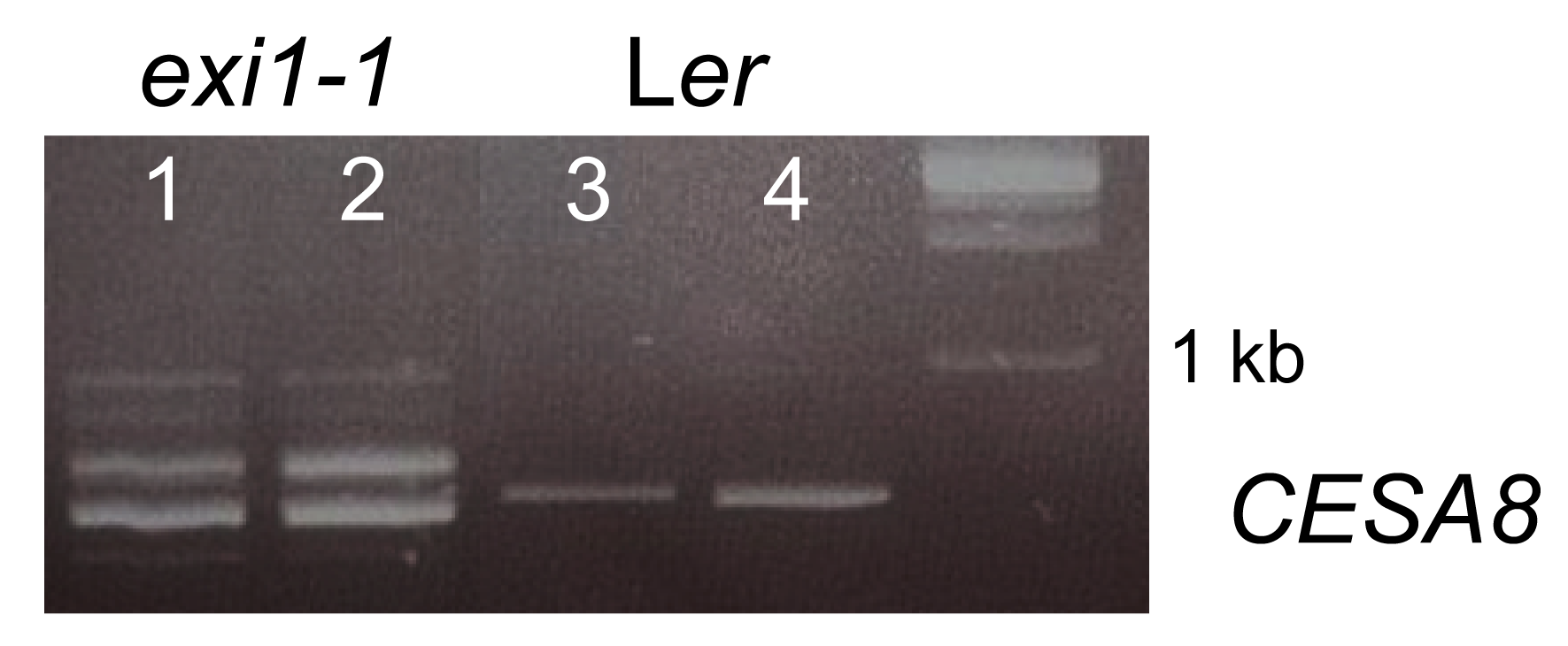

Supplement: Figure S2 — Agarose gel electrophoresis of RT-PCR amplification products obtained from CESA8 transcripts. RNA was extracted from exi1-1 (lanes 1, 2) and Ler (lanes 3, 4) rosettes collected 21 DAS, reverse transcribed and PCR amplified using the EXI1_3bF and EXI1_3R primers (Table S1). Lane 5: 1 kb DNA ladder (Invitrogen). (TIF) [file pone.0036500.s002.tif]

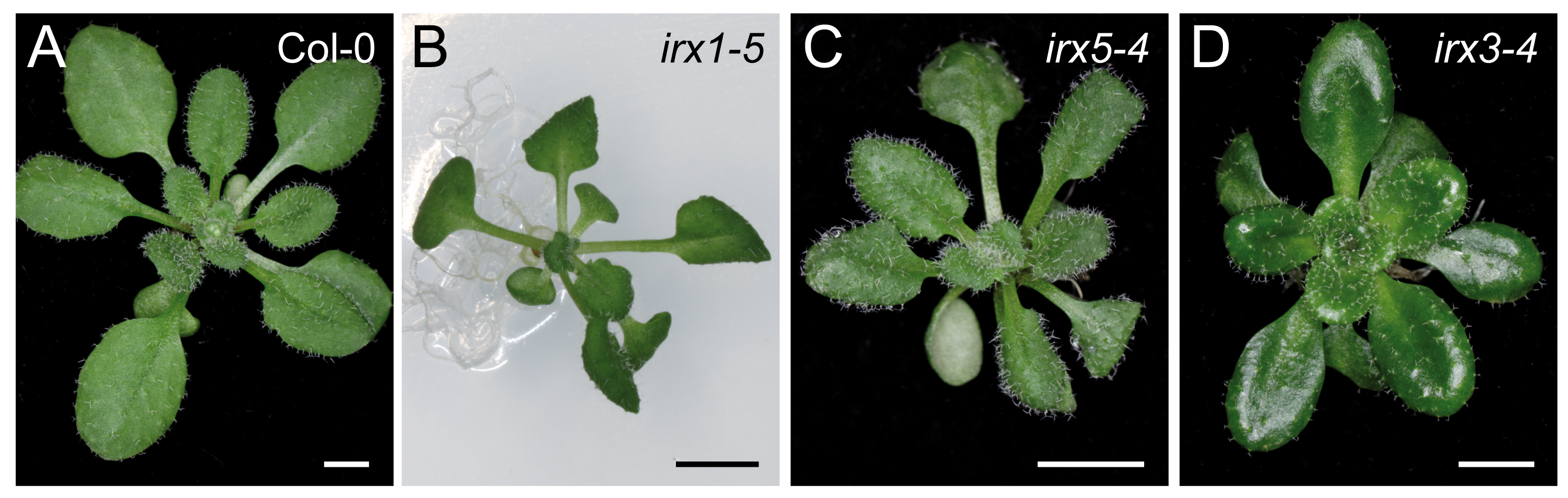

Supplement: Figure S3 — Mutants carrying T-DNA insertional alleles of the EXI genes. Rosettes are shown from (A) Col-0, (B) irx1–5 (Salk_026812), (C) irx5-4 (Salk_084627) and (D) irx3–4 (Salk_029940). Pictures were taken 21 DAS. Scale bars indicate 2 mm. (TIF) [file pone.0036500.s003.tif]
