# Supplementary material for: Cell Expansion-Mediated Organ Growth Is Affected by Mutations in Three EXIGUA Genes
Source: PLoS One. 2012 May 4;7(5):e36500. doi: 10.1371/journal.pone.0036500 (PMC3344895; doi:10.1371/journal.pone.0036500)
Supplement: Table S1 — Primers used in this work. (DOCX) [file pone.0036500.s004.docx]

**Table S1.** Primers used in this work.

| Primer | Sequence (5’ to 3’) |
| --- | --- |
| EXI1_1F | GAAAGAAACTTCTCAACTTCCG |
| EXI1_1R | ATGATCAACCGCATGATGATGAC |
| EXI1_2F | TCTCTGCCGTTTGTATTCTCAG |
| EXI1_2R | AGGTCTCTTCTCTCTAGAGACG |
| EXI1_3F | AACATTTGACCTGAAATGCTCTG |
| EXI1_3bF | CGTCTCTAGAGAGAAGAGACCT |
| EXI1_3R | TGATCGACCCGTAAATCCATCC |
| EXI1_4F | CATGTCATTAGCTGTGGATACG |
| EXI1_4R | GATGCTTTTGTTCATGGACTCTG |
| EXI1_5F | TTGTTTCTGTTCTTTATTGGAGC |
| EXI2_1F | GAAGCTTTCTTATGACACAAACC |
| EXI2_1R | ACCTCTCTTCTCTTGCCTCGC |
| EXI2_2F | GTGCAAACGCAAAATTTGAGACC |
| EXI2_2R | CATAACCCAACCTTCTTCAGGC |
| EXI2_3F | CAAAGATCGCAGAGCAATGAAGG |
| EXI2_3R | AGCTGATCATATCCTTCAAGCC |
| EXI2_4F | AGGAATCAAGAGCTTGTTCTCG |
| EXI2_4R | ATACCACAAAGGACAATGACGAC |
| EXI2_5F | TGGAAATCAGTTTACTGTATGCC |
| EXI2_5R | AAGTAGAAGCCCTGAATCACCG |
| EXI2_6F | TGTCTCAGCTCATCTCTTCGC |
| EXI5_1F | TTAGCTTGAAGGCAATGGAGAC |
| EXI5_1R | GATGATGGATATGGGTGCACAC |
| EXI5_2F | TAAGCATAAGCATTCTGCTGAGG |
| EXI5_2R | CTTTCAACGTGAAGTACATCTCC |
| EXI5_3F | ACGACGGTGCTTCAATGCTTAC |
| EXI5_3R | CGGAGGAACACCACCTTCTTC |
| EXI5_4F | GTCCATTCTTGCAGTAGATTTCG |
| EXI5_4R | AGATGTGTGCGTGTGTTTAGGC |
| EXI5_5F | CTAGTCTCTTCTTCATCTCAC |
